# Relatively Early and Late-Onset Neuromyelitis Optica Spectrum Disorder in Central China: Clinical Characteristics and Prognostic Features

**DOI:** 10.3389/fneur.2022.859276

**Published:** 2022-04-14

**Authors:** Jinbei Yu, Shuai Yan, Pengpeng Niu, Junfang Teng

**Affiliations:** ^1^Department of Neurology, The First Affiliated Hospital of Zhengzhou University, Zhengzhou, China; ^2^Department of Neurology, Affiliated Hospital of Hebei University, Baoding, China

**Keywords:** neuromyelitis optica spectrum disorder, relatively-late onset, disability, expanded disability status scale scores, disease course, annualized relapse rate, enlarged perivascular space

## Abstract

**Background:**

We aimed to analyze the clinical characteristics and prognostic features of Chinese patients with relatively late-onset neuromyelitis optica spectrum disorder (RLO-NMOSD>40 years of age at disease onset), compared with patients with relatively early onset NMOSD (REO-NMOSD, ≤ 40 years of age at disease onset).

**Methods:**

We retrospectively reviewed the medical records of patients with NMOSD in central China (with disease courses longer than 3 years) between January 2012 and January 2021. We further analyzed the clinical and prognostic differences between patients with REO-NMOSD and RLO-NMOSD.

**Results:**

A total of 71 patients were included in this study. The results showed that 39 (54.9%) of the patients had RLO-NMOSD. The patients with RLO-NMOSD had higher expanded disability status scale (EDSS) scores than patients with REO-NMOSD at the initial (5.0 *vs*. 3.0, *p* = 0.01), 3-month (4.0 *vs*. 2.5, *p* = 0.001), 1-year (4.0 *vs*. 2.5, *p* = 0.003), 3rd-year (3.5 *vs*. 3.0, *p* = 0.0017), and final follow-up (4.0 *vs*. 2.5, *P* = 0.002) time points. The EDSS scores of visual function were 2.0 (1.0–3.0) in REO-NMOSD and 3.0 (2.0–3.0) in RLO-NMOSD (*p* = 0.038) at the final follow-up time point. The locations of spinal cord lesions at transverse myelitis (TM) onset were prone to cervical cord in patients with REO-NMOSD. There were no between-group treatment differences. The risk of requiring a cane to walk (EDSS score of 6.0) increased as the age of disease onset increased: for every 10-year increase in the age of disease onset, the risk of needing a cane to walk increased by 65% [hazard ratio (*HR*) = 1.65, 95% *CI* 1.15–2.38, *p* = 0.007]. Another significant predictor identified in the multivariate analysis was annualized relapse rate (ARR) (*HR* = 2.01, 95% *CI* 1.09–3.71, *p* = 0.025). In addition, we observed a positive correlation between age at onset and EDSS scores at the final follow-up (Spearman's *r* = 0.426, *p* < 0.0001) time point. EDSS scores at different periods were significantly different between patients with RLO-NMOSD and REO-NMOSD with anti-aquaporin-4 (AQP4) IgG positive.

**Conclusion:**

The patients with RLO-NMOSD developed more severe disabilities than patients with REO-NMOSD at a variety of time periods. All of the patients may experience recurrent aggravated symptoms after their first year, with only patients with REO-NMOSD partly recovering from the 3rd year. The age at onset and ARR were the main predictors of outcomes.

## Introduction

Neuromyelitis optica spectrum disorder (NMOSD) is an autoimmune disease of the central nervous system that preferentially affects the optic nerve and spinal cord (1, 2). NMOSD was initially considered to be a special type of multiple sclerosis (MS). However, it is a different disease condition characterized by serum antibodies against anti-aquaporin-4 (AQP4)-IgG. NMOSD usually presents between the ages of 30 and 40 years (3).

The onset age of NMOSD is an important factor that can affect the disease relapse and severity (4, 5). Previous studies have reported that the rates of disability and mortality are different among early-onset NMOSD (EO-NMOSD <50 years at onset), late-onset NMOSD (LO-NMOSD, 50–70 years at onset), and very late-onset NMOSD (VLO-NMOSD, >70 years at onset) (5–9). The spinal cord may have increased the vulnerability to inflammation in LO-NMOSD, whereas the optic nerves are more susceptible in EO-NMOSD (7). Patients with LO-NMOSD had higher expanded disability status scale (EDSS) scores during remission and poorer prognoses, indicating that the age of onset is an important factor that affects the disease prognosis (6, 10, 11).

Currently, there are no clear definitions of the terms and cut-off values that are used for the age of onset. Most previous studies used a stratification of 50 years. Since the typical onset age is approximately 40 years, we compared the clinical characteristics of patients with NMOSD using an age stratification of 40 years (1, 3). We hoped that this age stratification would allow us to recommend suitable preventive treatments and improve the prognoses of patients. Most previous studies analyzed EDSS at onset and at the final follow-up time point (5, 7, 8, 12, 13). Although a few studies have analyzed EDSS throughout the long-term follow-up periods, some data suggest that the disease duration may vary between the two groups (5, 7, 13), while some studies only in patients with LO-NMOSD (12), or only in AQP4-Ab patients with NMOSD (8). We first performed a dynamic analysis of EDSS scores at the onset, 3-month, 1-year, 3-year, and final follow-up (~5-year) time points in patients with NMOSD who had disease courses >3 years. We hope that this approach will help us better understand the characteristics of the disease at different ages of onset, especially high paroxysmal ages.

Some studies have focused on differences in radiological features (i.e., brain lesions) between EO-NMOSD and LO-NMOSD (7, 8, 13–15). They found that NMO-typical brain lesions were common in patients with EO-NMOSD (7, 13), while non-specific white lesions were more frequently noted in patients with LO-NMOSD (8, 13). AQP4 is a water channel expressed in astrocyte endfeet, which wrap around capillaries (16). Thus, AQP4 lines central nervous system microvessels, pia, subpia, and perivascular spaces (17). In addition, they found that perivascular spaces (PVS) can be increased in MS (18). Enlarged PVS (EPVS) may serve as neurodegenerative markers in MS (18). However, there is little data on AQP4 and perivascular spaces in NMOSD (19). Here, we aimed to explore the characteristics of EPVS in REO-NMOSD and RLO-NMOSD.

## Methods

### Participants

This study was approved by the Medical Ethics Committee of the First Affiliated Hospital of Zhengzhou University (2021-KY-1103). We retrospectively screened the medical records of patients with NMOSD who came into contact with our healthcare system between January 2012 and January 2021 and had disease courses that were longer than 3 years. All patients were diagnosed with NMOSD based on the 2015 international diagnostic criteria for NMOSD (2, 20). In our study, MS had been ruled out according to the 2017 McDonald criteria (21). Patients with incomplete clinical data, who were lost to follow-up, had poor treatment compliance or had severe comorbidities, such as cerebrovascular diseases or tumors were excluded.

### Data Collection

Basic information, such as the age of onset, sex, disease duration, first symptoms (optic neuritis, myelitis, encephalopathy, and/or various combinations of these symptoms), EDSS scores were collected at baseline (at the time of the first attack) and at 3-month, 1-year, 3-year, and final follow-up time points. Additionally, we collected additional data, such as the time interval for the first relapse, the annualized relapse rate (ARR), the time to reach an EDSS score of 6.0, serum AQP4-IgG levels, cerebrospinal fluid (CSF) specific oligoclonal band (OCB), magnetic resonance images (MRIs) of the spinal cord and brain, visual EPVS rating scores in the basal ganglia (BG) and centrum semiovale (CS), treatments, and coexisting autoimmune disorders. Relapse was defined as an acute episode of neurologic symptoms that lasted over 24 h and occurred at least 30 days after the previous attack. An EDSS score of 6.0 was defined as “unilateral assistance needed to walk without wheelchair.”

Enlarged perivascular spaces are defined as linear-sharped or dot-like lesions with signal intensities similar to cerebrospinal fluid that are visible on T2-weighted MRI sequences (22). EPVS are distinguished from lacunes by having diameters lower than 3 mm and no hyperintense rims on the fluid-attenuated inversion recovery (FLAIR) sequences. We counted the number of EPVS in the CS and BG, and rated them with a validated 4-point semiquantitative scale (0 = no EPVS; 1 = 1–10 EPVS; 2 = 11–20 EPVS; 3 = 21–40 EPVS, and 4 ≥ 40 EPVS) (16). We included T1-weighted, T2-weighted, FLAIR, and Diffusion-Weighted Imaging (DWI) sequences. All MRI scans were evaluated by a neurologist who was blind to the clinical information.

Patients were divided into two groups: those with relatively-early onset NMOSD (REO-NMOSD; age at onset = 14–40 years) and those with relatively-late onset NMOSD (RLO-NMOSD; age at onset >40 years). Onset age refers to the age at which symptoms first appeared.

### Statistical Analysis

Statistical analyses were performed using SPSS 22.0. Continuous data were described as means ± standard deviation (SD) or as medians with inter-quartile range (IQR). Characteristics were compared between patients with LO-NMOSD and EO-NMOSD using χ^2^ (or Fisher's exact) tests for categorical data and Student's *t*-tests (or Wilcoxon rank-sum test) for continuous data. The Kaplan–Meier method was used to estimate the time between disease onset and reaching EDSS scores of 6.0/4.0. Predictive factors for disability were assessed using the Cox proportional hazards regression models. We compared the EPVS degree between the two groups using χ^2^ tests. The BG-EPVS and CS-EPVS degrees were dichotomized as high (score > 1) or low (score ≤ 1) (23, 24). The values of *p* < 0.05 were considered to be statistically significant.

## Results

### Demographic Data and Clinical Characteristics in REO-and RLO-NMOSD

**Figure 1:**
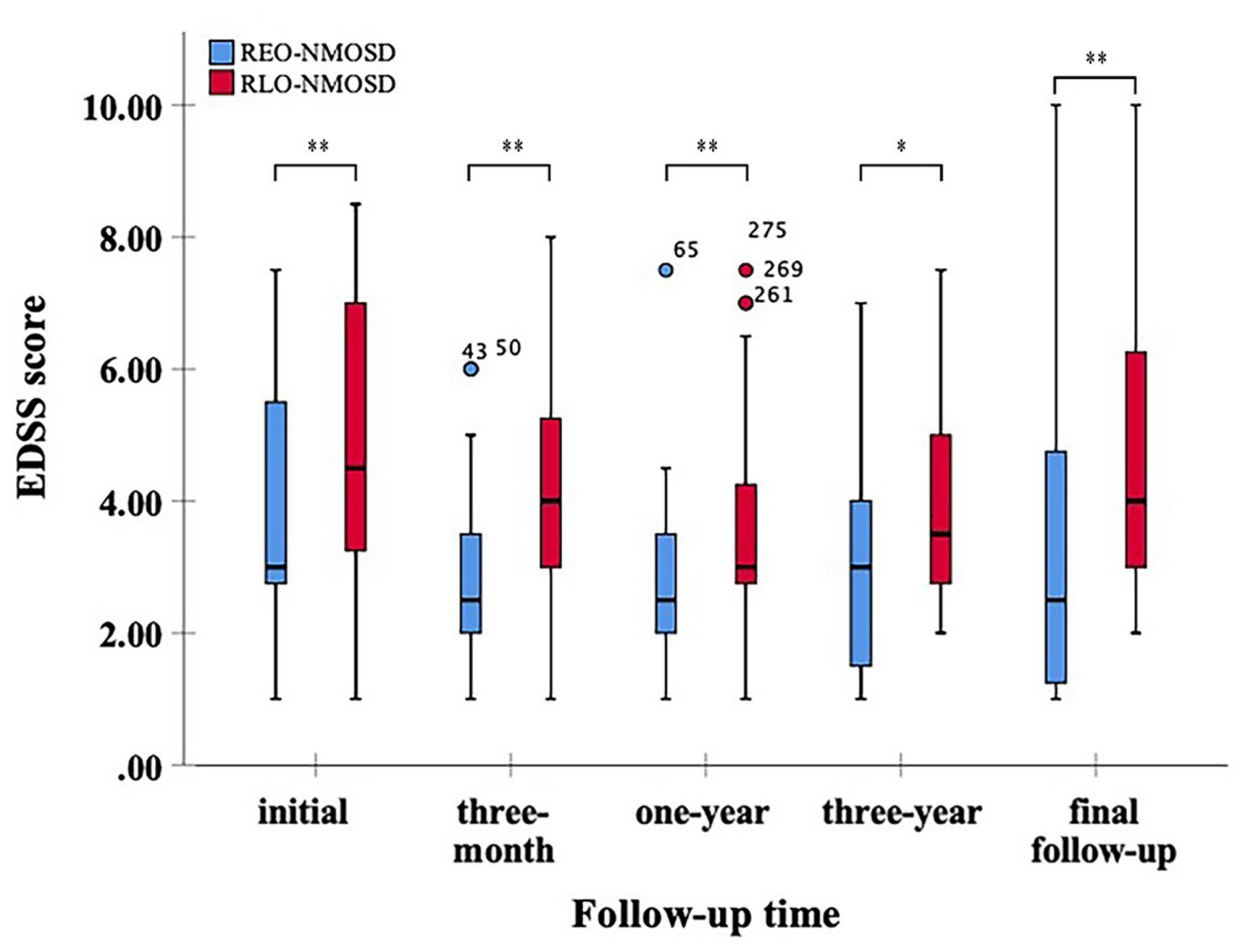
Expanded disability status scale (EDSS) scores at different time periods between patients with relatively early-onset neuromyelitis optica spectrum disorder (REO-NMOSD) and relatively late-onset neuromyelitis optica spectrum disorder (RLO-NMOSD) patients. (*p <0.05,**p <0.01).

**Figure 2:**
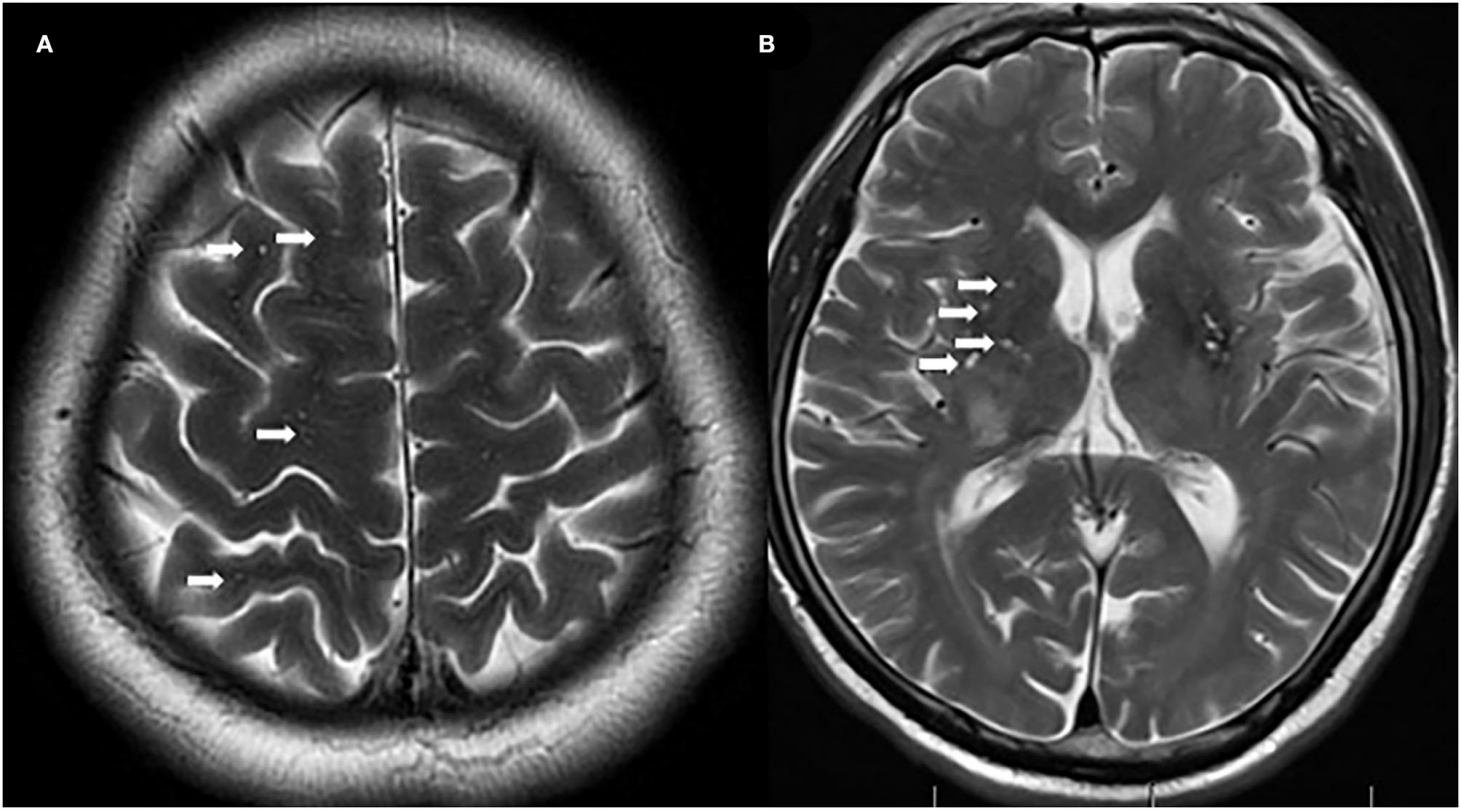
Representative examples of centrum semiovale-enlarged perivascular spaces (CS-EPVS) **(A)** and basal ganglia (BG)-EPVS **(B)**. Arrowheads point to individual EPVS. EPVS, enlarged perivascular spaces; BG, basal ganglia; CS, centrum semiovale.

A total of 71 patients (mean age: 39 ± 14 years) were enrolled in the study. The female:male ratio≈11:1. Among these patients, 32 patients were classified as having REO-NMOSD. The mean age of onset was 25.5 ± 6.7 years in the REO-NMOSD group and 50.8 ± 8.1 years in the RLO-NMOSD group. Compared with the RLO-NMOSD group, optic neuritis (ON) was more frequently observed at the time of initial attack in the REO-NMOSD group (43.8 vs. 30.8%), but there were no statistically significant differences. There were no significant between-group differences in the number of patients with at least one ON (REO-NMOSD 21/32, 65.6%; RLO-NMOSD 26/39, 66.7%, *p* = 0.926). The involvements of transverse myelitis (TM) were more common in the RLO-NMOSD group (48.7 vs. 25.0%, *p* = 0.041). Overall, the chance of having the initial symptoms of area postrema syndrome (APS) was significantly higher in the REO-NMOSD group compared with the RLO-NMOSD group (6/32 vs. 1/39, *p* = 0.04). APS was more common in the REO-NMOSD group, while TM was more frequent in the RLO-NMOSD group. Moreover, we found that EDSS scores at the initial and 3-month time points were significantly higher in the REO-NMOSD group (Table 1). ARR and duration to relapse were not significantly different between the groups. EDSS scores in the RLO-NMOSD group were significantly higher at the 1-year time point (*p* = 0.003). At the 3-year time point, the EDSS scores were 3.0 (1.5–4.0) for the REO-NMOSD group and 3.5 (2.9–5.1) for the RLO-NMOSD group (*p* = 0.017). This difference persisted to the last follow-up time point (*p* = 0.002; Figure 1). We further compared the EDSS scores of visual functioning in patients with ON occurrence. EDSS scores were not significantly different between the two groups at initial attack (*p* = 0.466). The nadir EDSS scores showed no between-group differences (*p* = 0.716). Surprisingly, however, patients with RLO-NMOSD had more serious visual sequelae than patients with REO-NMOSD (Table 1). EDSS scores averaged 2.0 (1.0–3.0) in the REO-NMOSD group and 3.0 (2.0–3.0) in the RLO-NMOSD group at the final follow-up time period (*p* = 0.038). No significant between-group differences were found in the rates of coexisting autoimmune disorders (43.8 vs. 30.8%, *p*>0.05). There were no differences in treatments between the two groups (Table 1). Serum AQP4-IgG was observed in a large proportion of patients with REO-NMOSD (25, 78.1%) as well as patients with RLO-NMOSD (26, 66.7%). In serum AQP4-IgG negative patients, CSF-specific OCB markers were negative. Representative examples of EPVS are illustrated in Figure 2. There were no differences in BG-EPVS and CS-EPVS scores between the two groups (Table 1). For spine MRI findings, 29 patients (29/32, 90.6%) in the REO-NMOSD group and 37 patients (37/39, 94.9%) in the RLO-NMOSD group suffered from at least one TM disease. The median lesion length was 6.0 vertebral segments (IQR, 4.0–7.4) in the REO-NMOSD group and 7.0 (4.5–11.5) in the RLO-NMOSD group at TM onset, which was not a significant difference (Supplementary Table 1). The longest segments during disease course showed no differences between the two groups (*p* = 0.521). The locations of spinal cord lesions included the cervical cord, thoracic spine, lumbar spine, and/or both. The locations of spinal cord lesions at TM onset were prone to cervical cord in patients with REO-NMOSD (10/29 vs. 4/37, *p* = 0.032). There were no between-group differences in the distribution of longest lesion locations (Supplementary Table 1).

**Table 1:** The demographic and clinical characteristics of patients with neuromyelitis optica spectrum disorder (NMOSD) according to age group (<40 or ≥40 years).

**Characteristic**	**REO-NMOSD**	**RLO-NMOSD**	***P*-value**
	**(*n* = 32)**	**(*n* = 39)**	
Female: male (ratio)	29:3 (9.7:1)	36:3 (12:1)	1.000
Age at onset, years, IQR	25 (21.3–31.8)	50 (45.0–56.0)	NA
Disease duration (months) IQR	56.5 (43.2–81.0)	63 (46–75)	0.977
**Onset attack type**, ***n*** **(%)**			
Optic neuritis	14 (43.7%)	12 (30.8%)	0.259
Transverse myelitis only	8 (25.0%)	19 (48.7%)	0.041
Area postrema syndrome	6 (18.8%)	1 (2.6%)	0.041
Other	4 (12.5%)	7 (17.9%)	0.763
Time to first relapse, months	7.0 (3.0–16.8)	10.5 (5.0–16.8)	0.339
Annualized relapse rate	1.0 (0–1.0)	1.0 (0–1.0)	0.772
**EDSS score, IQR**			
At initial	3.0 (2.6–5.8)	5.0 (3.0–7.0)	0.010
Third month follow-up	2.5 (2.0–3.5)	4.0 (3.0–5.5)	0.001
First year follow-up	2.5 (2.0–3.5)	3.0 (2.5–4.5)	0.003
Third year follow-up	3.0 (1.5–4.0)	3.5 (2.9–5.1)	0.017
Latest follow-up	2.5 (1.1–4.9)	4.0 (3.0–6.9)	0.002
**EDSS score of visual acuity, IQR**			
At initial	3.0 (2.0–3.0)	3.0 (2.0–3.0)	0.466
Nadir	3.0 (2.5–3.5)	3.0 (2.0–4.0)	0.716
Final follow-up	2.0 (1.0–3.0)	3.0 (2.0–3.0)	0.038
Time to EDSS score 6, weeks (IQR)	146 (5.5–273)	74.0 (2.0–196.0)	0.116
Coexisting autoimmune disorders	14 (43.8%)	12 (30.8%)	0.259
**Immunosuppressant therapy (IST)**	28 (87.5%)	34 (87.2%)	1.000
Time from onset to IST	8 (4–28.5)	9.5 (1–22.2)	0.644
Azathioprine	14	24	0.135
Mycophenolate mofetil	11	8	0.189
Other (MTX/RTX)	3	2	0.652
Without IST	4	5	1.000
**Serostatus**, ***n*** **(%)**			
AQP4-IgG positive	25 (78.1%)	26 (66.7%)	0.302
**CS EPVS**, ***n*** **(%)**			0.329
High degree-CS EPVS (score > 1)	16 (50.0%)	24 (61.5%)	
Low degree-CS EPVS (score ≤ 1)	16 (50.0%)	17 (38.5%)	
**BG EPVS**, ***n*** **(%)**			0.086
High degree-BG EPVS (score > 1)	7 (21.9%)	16 (41.0%)	
Low degree-BG EPVS (score ≤ 1)	25 (78.1%)	23 (59.0%)	

*MTX, methotrexate; RTX, rituximab; CS, centrum semiovale; BG, basal ganglia*.

### Predictors of the Development of Disability in REO-and RLO-NMOSD

We investigated whether age, disease duration, EDSS scores at the time of disease onset, serostatus, initial attack type, ARR, and immunosuppressant therapies affected the development of disabilities in NMOSD. We found that the risk of needing a cane to walk (EDSS score of 6.0) increased with an older age of disease onset. For every 10-year increase, the risk of needing a cane to walk increased by 65% [hazard ratio (*HR*) = 1.65, 95% *CI* 1.15–2.38, *p* = 0.007]. Another significant predictor identified in the multivariate analysis was ARR (*HR* = 2.01, 95% *CI* 1.09–3.71, *p* = 0.025).

**Figure 3:**
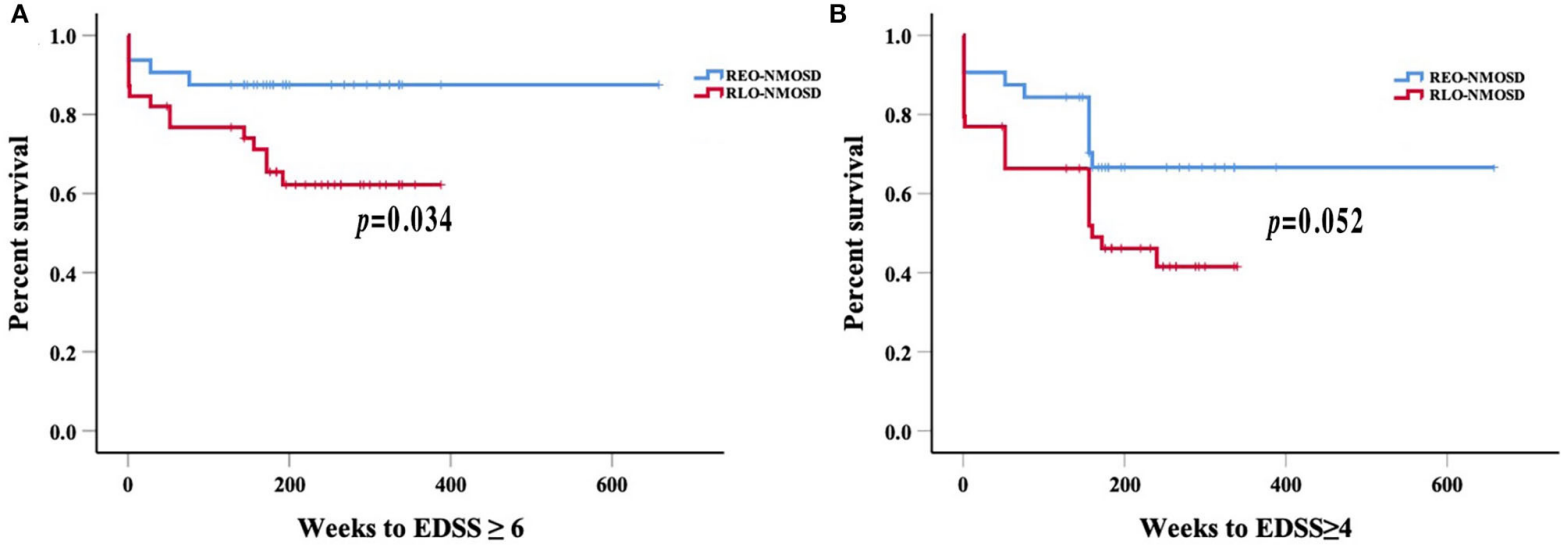
Weeks from onset to reach the EDSS scores of 6.0 and 4.0, stratified by the age of disease onset: **(A)** Patients with RLO-NMOSD reached EDSS scores of 6.0 sooner than patients with REO-NMOSD (*p* = 0.034); **(B)** Time to EDSS score of 4.0 between the two groups (*p* = 0.052).

**Figure 4:**
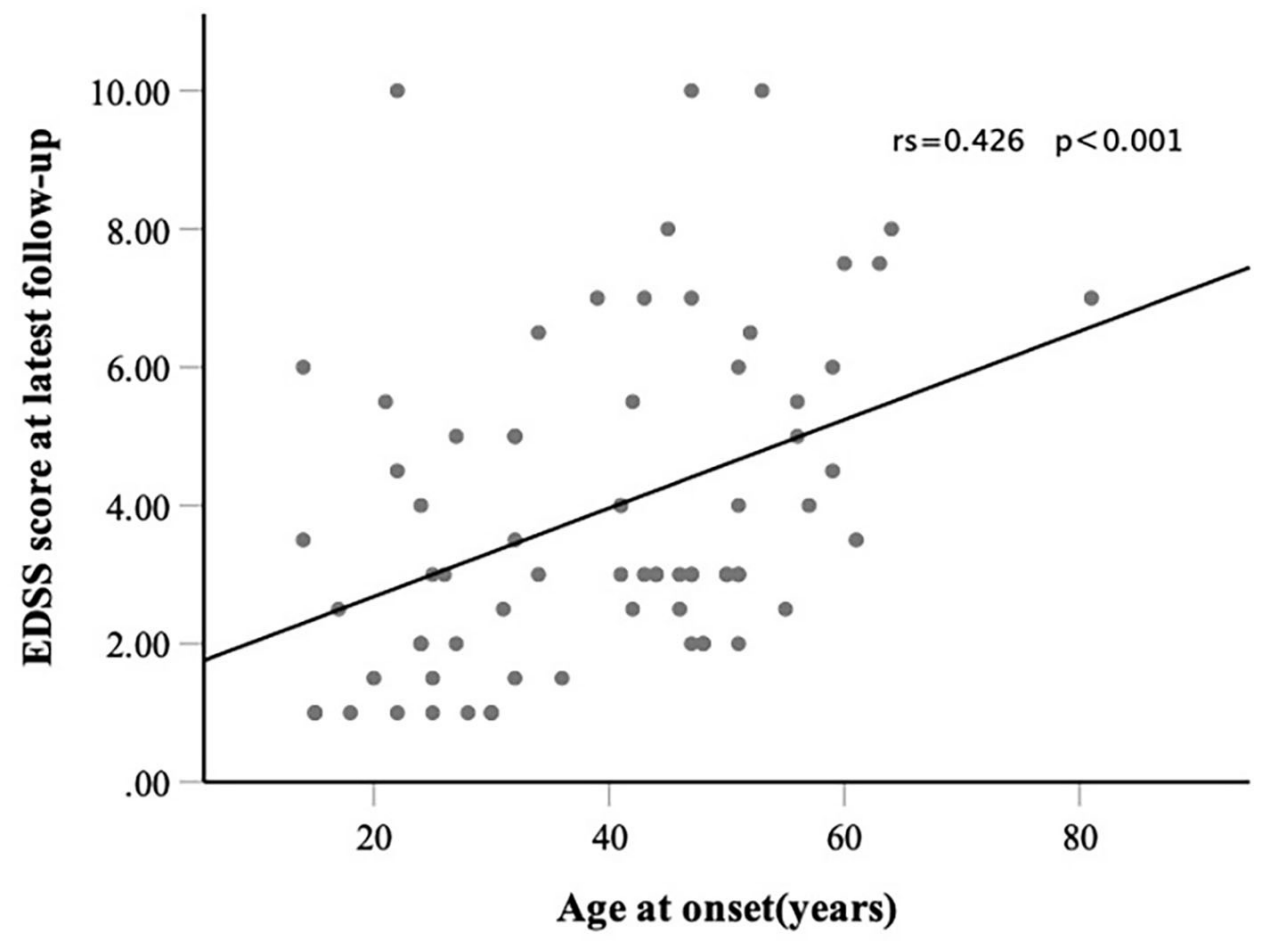
Correlation between onset age and EDSS at the final follow-up time point.

Consistent with reaching an EDSS score of 6.0, we found that for every 10-year increase in the age of disease onset, the risk of reaching an EDSS score of 4.0 increased by 51.9% (*HR* 1.51, 95% *CI* 1.14–2.02, *p* = 0.004). ARR was another significant predictor (*HR* 1.71 95% *CI* 1.06–2.74, *p* =0.027). The risk of reaching an EDSS score of 6.0 with RLO-NMOSD was three times higher than with EO-NMOSD (*HR* 3.06, 95% *CI* 1.01–9.29, *p* = 0.049). The time to reach the EDSS scores of 6.0 and 4.0 in the two groups was shown in Figures 3A,B. EDSS score at the final follow-up time point was also significantly correlated with the age of disease onset (Figure 4).

### Demographic Data and Clinical Characteristics in REO-and RLO-NMOSD With AQP4-Ab Positive

**Figure 5:**
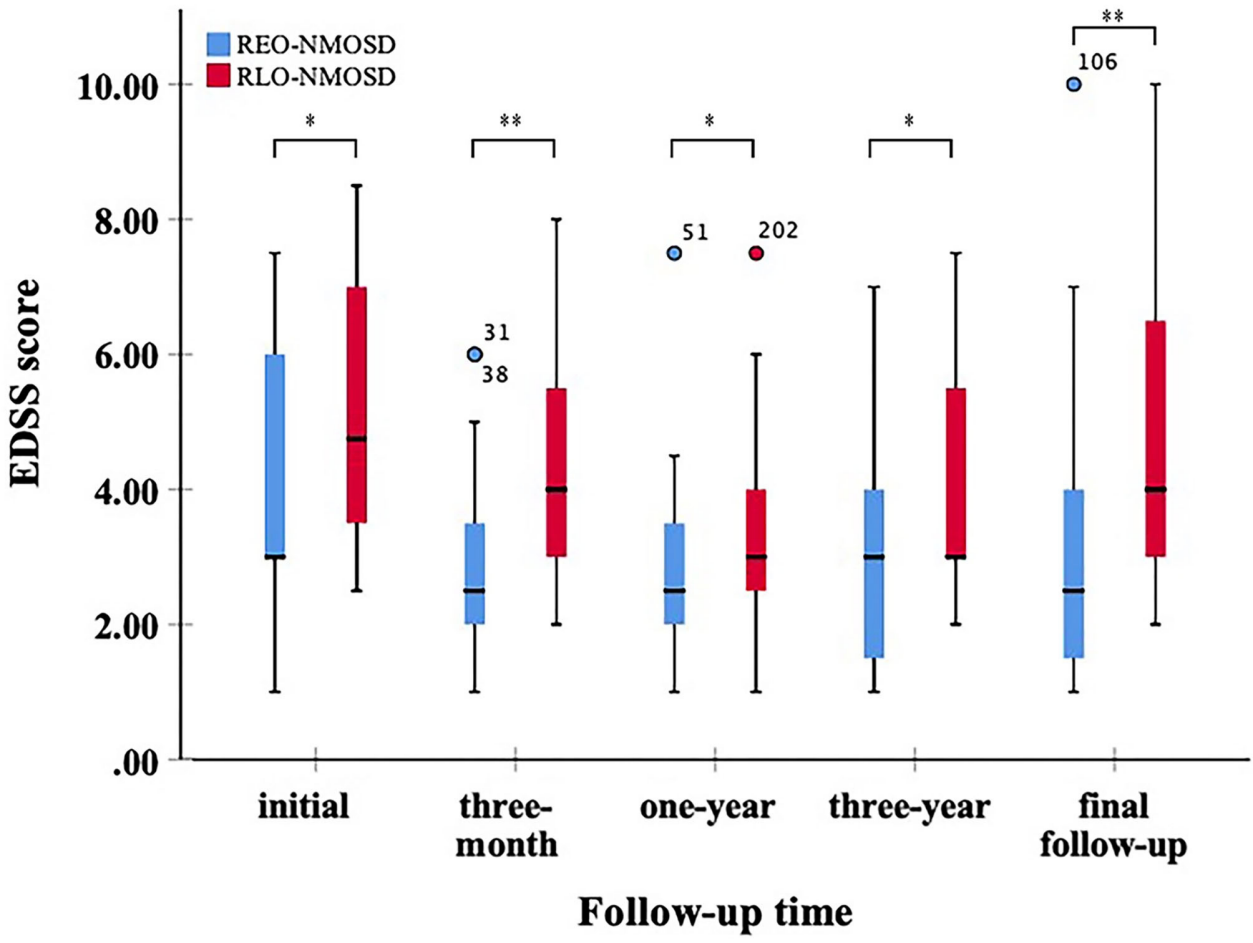
Expanded disability status scale scores at different points between patients with REO- and RLO-NMOSD with AQP4-Ab positive (*p <0.05,**p <0.01).

There were 51 (70.83%) patients whose serum AQP4-Ab was positive. Among these, 25 patients (49.02%) were REO-NMOSD, and 26 patients (50.98%) were RLO-NMOSD (Table 2). In AQP4-Ab positive NMOSD, RLO-NMOSD patients' EDSS scores were significantly higher than the score of patients with REO-NMOSD at the time of disease onset (*p* = 0.039); Figure 5, and 3 months after treatment (*p* = 0.004). EDSS scores at the 1-year time points were also different between groups (*p* = 0.032). At the 3-year time points, the average EDSS score was 3.0 (1.0–4.0) in the REO-NMOSD group and 3.0 (2.88–5.63) in the RLO-NMOSD group (*p* = 0.046). Differences in EDSS scores also persisted at the final follow-up time point (*p* = 0.008). Although the proportion of EDSS scores >6 at the final follow-up time point was higher in the RLO-NMOSD group (34.6 *vs*. 16.0%), it did not differ significantly between the two groups (*p* = 0.199). The percentage of EDSS scores >4.0 was significantly different between the two groups *(p* = 0.032). The degrees of BG-EPVS and CS-EPVS showed no differences between the two groups (Table 2). Other factors, such as sex, ARR, type of onset, coexisting autoimmune disorders, and chronic immunosuppressant therapy, were not significantly different between the two groups.

**Table 2:** The comparison of demographic and clinical features between patients with REO-NMOSD and RLO-NMOSD with AQP4-IgG.

**Characteristic**	**REO-NMOSD**	**RLO-NMOSD**	**P-value**
	**(*n* = 25)**	**(*n* = 26)**	
Female: male (ratio)	24:1 (24:1)	12:1 (24:2)	1.000
Age at onset, years, mean (SD)	26.9 (6.6)	51.9 (8.5)	NA
Disease duration (months) IQR	67.0 (44.5–82.5)	62.5 (44.8–76.3)	0.644
**Onset attack type**, ***n*** **(%)**			
Optic neuritis	10 (40.0%)	10 (38.5%)	0.910
Transverse myelitis only	8 (32.0%)	10 (38.5%)	0.447
Area postrema syndrome	4 (16.0%)	1 (3.8%)	0.191
Other	3 (12.0%)	5 (19.2%)	0.703
Time to first relapse, months	9.0 (3.0–21.0)	9.0 (5.0–20.5)	0.634
Annualized relapse rate	1.0 (0.0–1.0)	1.0 (0.0–1.0)	0.843
**EDSS score, IQR**			
At initial	3.0 (2.8–6.3)	4.8 (3.5–7.0)	0.039
Third month follow-up	2.5 (2.0–4.0)	4.0 (3.0–5.5)	0.004
First year follow-up	2.5 (1.8–3.5)	3.0 (2.5–4.0)	0.032
Third year follow-up	3.0 (1.5–4.0)	3.0 (2.9–5.6)	0.046
Latest follow-up	2.5 (1.5–4.5)	3.8 (3.0–6.6)	0.010
EDSS score≥6 *n* (%)	4 (16.0%)	9 (34.6%)	0.127
EDSS score≥4 *n* (%)	7 (28.0%)	15 (57.7%)	0.032
Coexisting autoimmune disorders	13 (52.0%)	10 (38.5%)	0.331
**Immunosuppressant therapy (IST)**	22 (88.0%)	23 (88.5%)	1.000
Time from onset to IST	9.5 (2.5–25.5)	9.0 (1.0–28.0)	0.663
Azathioprine	12 (48.0%)	15 (57.7%)	0.488
Mycophenolate mofetil	9 (36.0%)	7 (26.9%)	0.485
Other (MTX/RTX)	1 (4.0%)	1 (3.8%)	0.745
Without IST	3 (12.0%)	3 (11.4%)	1.000
**CS EPVS**, ***n*** **(%)**			0.867
High degree-CS EPVS (score > 1)	15 (60.0%)	15 (57.7%)	-
Low degree-CS EPVS (score ≤ 1)	10 (40.0%)	11 (42.3%)	-
**BG EPVS**, ***n*** **(%)**			0.406
High degree-BG EPVS (score > 1)	6 (24.0%)	9 (34.6%)	-
Low degree-BG EPVS (score ≤ 1)	19 (76.0%)	17 (65.4%)	-

*MTX, methotrexate; RTX, rituximab; CS, centrum semiovate; BG, basal ganglia*.

## Discussion

All participants enrolled in our study had long disease courses and were followed-up for at least 3 years, which is different than the varied lengths of follow-up in previous studies (5, 7, 13). Patients with RLO- and REO-NMOSD had similar sex ratios, treatments, and serologic features. However, patients with RLO-NMOSD had higher EDSS scores than patients with REO-NMOSD at various time points. These patients might partly recover after the acute phase but then experience symptom aggravation in the following years. They had more serious visual sequelae than patients with REO-NMOSD. A similar phenomenon was noted in AQP4-Ab positive patients with NMOSD. EDSS at the final follow-up time point was significantly correlated with the age of disease onset. The risk of needing a cane to walk was also correlated with onset age and ARR.

The proportion of female in our study is higher than previous studies, which were about (6–9):1 (1, 7, 13, 15, 25). It may be related to relapsing NMOSD being prone to female (3). Another factor to consider is that a relatively small sample was enrolled in our singer center. Previous studies have identified the effect of age at the disease onset on motor disability and prognosis, suggesting that older patients are more susceptible to disability over short-time courses (5, 7, 8, 13). Previous work showed that the late age onset (i.e., >50 years) was associated with higher EDSS scores at remission, the higher rates of mortality, and the higher rates of motor disability. Pediatric patients with NMOSD predominantly present with ON (7, 26). In some previous LO-NMOSD cohorts, an average EDSS score of 3.0 (2.0–5.0) was reported at the final follow-up time point (7, 12, 13). However, the follow-up time varied between groups (7, 12, 13, 27). The onset age of NMOSD usually presents in the 40 years, especially in Blacks and Asians (Blacks: ~28–33 years, Asians: 35–40 years, vs. Whites: 44 years) (1, 3, 25–28). We aimed to investigate the characteristic and prognostic features of NMOSD under the stratification of 40 years old. Previous studies that focused on AQP4-Ab-positive patients found that the LO-NMOSD group had severer disability at 2 or 5 years than patients with EO-NMOSD (27). Here, we selected patients who had disease courses more than 3 years and found that EDSS scores were significantly higher in patients with RLO-NMOSD at different time periods (the onset, 3-month, 1-year, 3-year, and the latest follow-up time points). Patients can partly recover from the first episode after treatment, while could be worse after 3 years. Only patients with REO-NMOSD can partly recover again from 3 years. These results had not been reported in previous studies. The latest EDSS score was 2.5 (1.1–4.9) in the REO-NMOSD group and 4.0 (3.0–6.9) in the RLO-NMOSD group after about 5 years of follow-up. Unlike the previous study (29), the severity of visual acuity showed no between-group differences no matter at onset or the nadir status. However, patients with RLO-NMOSD suffered from more serious sequelae than patients with REO-NMOSD. Additionally, in AQP4-Ab positive patients with NMOSD who had the disease for at least 3 years, the RLO-NMOSD group had higher EDSS scores than the REO-NMOSD group at different time points. The patients with REO-NMOSD who were AQP4-Ab positive could partly recover after the 3rd year, but the patients with RLO-NMOSD could not. We further validated that the onset age was a crucial determinant of disease severity. In addition, the risk of needing a cane to walk increased with the increased age at disease onset and ARR.

Neuromyelitis optica spectrum disorder is an astrocytopathy that mainly affects the spinal cord, optic nerves, and area postrema. Previous studies showed that TM at the onset was more frequently observed in the LO-NMOSD group than in the EO-NMOSD group, while the proportion of patients with at least one ON was higher in the EO-NMOSD group (7, 8, 26, 30, 31). In our study, we found that TM was more common in patients with RLO-NMOSD, while the proportion of ON showed no significant differences between the two groups. Additionally, the media spine cord lesion length at initial attack or the longest segments during disease course also had no significant differences between the two groups. Simialr to previous studies (9, 32), we found that the location of spine cord lesions were mainly at cervical cord and/or thoracic cord. However, patients with REO-NMOSD were more likely to have cervical cord involvement than patients with RLO-NMOSD at TM initial attack. APS is one of the core clinical symptoms of NMOSD (20), and is characterized by intractable nausea, vomiting, and hiccups. APS occurred during disease onset in 7.1–10.3% of patients with NMOSD (33). Here, we found that APS incidence in patients with REO-NMOSD was significantly higher than in patients with RLO-NMOSD. The disability causes poor recovery from attacks (34).

Brain MRI lesions identified in NMOSD included typical brain lesions, peri ependymal lesions surrounding the third ventricles and lateral ventricles, and extensive hemispheric lesions (7, 8, 13). Mao et al. found that the differing characteristics of brain lesions between patients with EO-NMOSD and LO-NMOSD may reflect inflammatory processes (8). AQP4 is a perivascular astrocyte channel protein that regulates glymphatic function (35) and facilitates CSF flow. Convective interstitial fluid (ISF) propels waste products toward veins where they enter the PVS for efflux out of the central nervous system (36). The PVS is lined by astrocytic endfeet which have AQP4 water channels on the exteriors that abutting abluminal vessel walls at the inner boundaries (37). PVS visibility increases with age (i.e., is strongest in the BG) (16, 38, 39). Additionally, it is associated with hypertension and inflammation (16, 38, 40). In this study, we compared the degrees EPVS between patients with REO-NMOSD and RLO-NMOSD. We found that the degrees of CS-EPVS were not significantly different between the two groups, which was the same as in BG-EPVS. In AQP4-Ab positive NMOSD, EPVS degrees were also similar between the REO-NMOSD and RLO-NMOSD groups. In the future, we hope to compare EPVS in NMOSD patients with EPVS in a matched healthy control group and to investigate the PVS kinetics in the two groups.

Our study has limitations: it is a retrospective study and contained a small number of patients from a single center.

In conclusion, for patients with REO- and RLO-NMOSD with disease courses longer than 3 years, those with disabilities can partly recover from their initial attacks during the first year. Patients with RLO-NMOSD become worse throughout time until the final follow-up time points, while patients with REO-NMOSD partly recovered after their third year. Patients with RLO-NMOSD had a worse final visual acuity than patients with REO-NMOSD, while the lengths of spinal cord involvement showed no difference between the two groups. Besides, patients with REO-NMOSD were more likely to have cervical cord involvement than patients with RLO-NMOSD at TM onset. Similar features were reflected in AQP4-Ab positive NMOSD. The age of onset and ARR were the main predictors for disability.

## Data Availability Statement

The original contributions presented in the study are included in the article/Supplementary Material, further inquiries can be directed to the corresponding author.

## Ethics Statement

The studies involving human participants were reviewed and approved by Medical Ethics Committee of the First Affiliated Hospital of Zhengzhou University (2021-KY-1103). Written informed consent from the participants' legal guardian/next of kin was not required to participate in this study in accordance with the national legislation and the institutional requirements.

## Author Contributions

JY conceived and designed the study, involved in the acquisition of data, and writing the original draft. SY contributed in the data analysis and editing of the review. PN and JT provided critical revisions to the article. All authors contributed to the article and approved the submitted version.

## Funding

This work was supported by the Program of Science and Technology Development of Henan Province of China [NO. 2018020108].

## Conflict of Interest

The authors declare that the research was conducted in the absence of any commercial or financial relationships that could be construed as a potential conflict of interest.

## Publisher's Note

All claims expressed in this article are solely those of the authors and do not necessarily represent those of their affiliated organizations, or those of the publisher, the editors and the reviewers. Any product that may be evaluated in this article, or claim that may be made by its manufacturer, is not guaranteed or endorsed by the publisher.
