# Supplementary material for: Relatively Early and Late-Onset Neuromyelitis Optica Spectrum Disorder in Central China: Clinical Characteristics and Prognostic Features
Source: Front Neurol. 2022 Apr 14;13:859276. doi: 10.3389/fneur.2022.859276 (PMC9046694; doi:10.3389/fneur.2022.859276)
Supplement: Supplementary file 1 [file Table_1.pdf]

Supplementary Table1 Spinal cord MRI findings in patients with NMOSD-TM according to age group(< 40 or ≥40 years)

Spinal cord MRI imaging	REO-NMOSD (n=29)	RLO-NMOSD (n=37)	*P* value
Length of lesion, vertebral segments (IQR)			
At initial attack	6.0 (4.0-7.0)	7.0 (4.5-11.5)	0.203
Longest segments	7.0 (5.0-13.5)	9.0 (6.0-13.0)	0.521
Location of lesion (at initial attack), n (%)			
Cervical cord	10(34.5%)	4 (10.8%)	0.032
Thoracic cord	5 (17.2%)	13 (35.1%)	0.164
Cervical and Thoracic cord	12 (41.4%)	19 (51.4%)	0.464
Other (Lumbar involved)	2 (6.9%)	1 (2.7%)	0.829
Location of lesion (longest segments), n (%)			
Cervical cord	6 (20.7%)	2 (5.4%)	0.131
Thoracic cord	3 (10.3%)	11 (29.7%)	0.056
Cervical and Thoracic cord	17 (58.6%)	23 (62.2%)	0.770
Other (Lumbar involved)	3 (10.3%)	1 (2.7%)	0.440

MRI, magnetic resonance imaging; NMOSD, neuromyelitis optica spectrum disorders;

NMOSD-TM: NMOSD patients who had at least one transverse myelitis (TM)；

REO-NMOSD: relatively-early onset NMOSD

RLO-NMOSD: relatively-late onset NMOSD
